# A putative curved DNA region upstream of *rcsA* in *Escherichia coli* plays a key role in transcriptional regulation by H‐NS

**DOI:** 10.1002/2211-5463.12348

**Published:** 2018-06-26

**Authors:** Shanmugaraja Meenakshi, Maruthan Karthik, M. Hussain Munavar

**Affiliations:** ^1^ Department of Molecular Biology School of Biological Sciences Centre for Advanced Studies in Functional and Organismal Genomics Madurai Kamaraj University [University with Potential for Excellence] Madurai India

**Keywords:** curved DNA, H‐NS, *rcsA*

## Abstract

It is well established that in *Escherichia coli*, the histone‐like nucleoid structuring (H‐NS) protein also functions as negative regulator of *rcsA* transcription. However, the exact mode of regulation of *rcsA* transcription by H‐NS has not been studied extensively. Here, we report the multicopy effect of dominant‐negative *hns* alleles on the transcription of *rcsA* based on expression of *cps‐lac* transcriptional fusion in ∆*lon*, ∆*lon rpoB12*, ∆*lon rpoB77* and *lon* ^+^ strains. Our results indicate that H‐NS defective in recognizing curved DNA fails to repress *rcsA* transcription significantly, while nonoligomeric H‐NS molecules still retain the repressor activity to an appreciable extent. Together with bioinformatics analysis, our study envisages a critical role for the putative curved DNA region present upstream of *rcsA* promoter in the transcriptional regulation of *rcsA* by H‐NS.

**Abbreviations**
Ces: capsule expression suppression
Cps: capsular polysaccharide
H‐NS: histone‐like nucleoid structuring
LB medium: Luria–Bertani medium
OD: optical density
RcsA: regulator of capsule synthesis A
rpoB: RNA polymerase beta subunit

In *Escherichia coli*, the genetic material is organized in the form of nucleoid and the DNA‐binding proteins such as histone‐like proteins serve as a dynamic scaffold for nucleoid organization 1, 2, 3, 4. The histone‐like nucleoid structuring (H‐NS, previously denoted as H1) protein of *E. coli* is one of the major components of the nucleoid. *hns* gene was identified by Pon *et al*. 5, and it maps at 27 min of *E. coli* chromosome. H‐NS protein comprises 137 amino acids with 15.5 kDa molecular weight. Although initial studies suggested that H‐NS is involved only in the organization of chromosome, the identification that H‐NS has higher propensity to bind to DNA, especially the AT‐rich sequences, clearly indicated the regulatory function associated with H‐NS 6, 7, 8. H‐NS was found to affect gene expression in a number of different ways, and it has been reported that expression of over 5% of the *E. coli* genes is affected in *hns* mutant 9, 10, 11.

H‐NS binding does not seem to occur with any obvious sequence specificity 12, 13. Different mechanisms for transcriptional regulation by H‐NS have been proposed; the most accepted models are as follows: H‐NS might indirectly regulate initiation by binding to region distal from the promoter which causes change in supercoiling that in turn affects the supercoiling‐sensitive promoters; and H‐NS can also directly inhibit transcription by preferential binding to the promoter region. Many of the preferred H‐NS binding sites contain an A/T‐rich region, suggesting that a sequence‐induced curvature is causing the preferential binding 14, 15, 16, 17, 18. Studies on structural aspects of H‐NS revealed that H‐NS is comprised of a C‐terminal DNA‐binding domain and a coiled‐coil N‐terminal domain that mediates oligomerization, forming higher‐order homomeric or heteromeric complexes. At least two dimerization sites have been identified that allow H‐NS to form higher‐order oligomers 19, 20, 21, 22. The oligomerization and DNA‐binding domains are joined via a flexible linker.

H‐NS itself acts as a repressor for its own promoter, and apart from H‐NS, StpA, Fis and CspA also play a role in the regulation of H‐NS expression 23, 24. There is also a post‐transcriptional negative regulatory mechanism which involves a small RNA called DsrA and an RNA‐binding chaperon protein called Hfq 25, 26. The expression of H‐NS is also increased by an unknown mechanism during growth at elevated hydrostatic pressure. H‐NS and other nucleoid‐associated proteins can recognize horizontally acquired DNA and transcriptionally silence it through xenogeneic silencing under environmental conditions that do not require expression of horizontally acquired genes 27, 28. In addition to its role in nucleoid architecture, H‐NS plays a pleiotropic role in bacterial response to environmental stimuli such as starvation and changes in pH, temperature and osmolarity 29, 30, 31.

Very recently, we have reported the suppression of overexpression of genes implicated in colanic acid capsular polysaccharide (Cps) synthesis in ∆*lon* mutant of *E. coli* by two novel *rpoB* mutations, namely *rpoB12* and *rpoB77*. Genetic and molecular analyses clearly showed that downregulation of *rcsA* transcription is the primary reason for the elicitation of this capsule expression suppression (Ces) phenotype by these two *rif* alleles. Furthermore, our study clearly indicated that the presence of functional H‐NS is mandatory for both the *rpoB* mutations to function as capsule expression suppressors in the ∆*lon* strain of *E. coli* 32. Sledjesky and Gottesman 25 have shown that H‐NS functions as a repressor for *rcsA* transcription, and their study also revealed the involvement of a small RNA, namely DsrA located downstream of *rcsA*, in the regulation of *rcsA* by H‐NS. DsrA binds to H‐NS and thereby inhibits the action of H‐NS on *rcsA* transcription. However, the mode of binding and the exact binding region for H‐NS in the promoter region of *rcsA* have not been reported so far. They have suggested that the upstream region of *rcsA* promoter might possess bending/curved DNA region 25. In our earlier study, we have provided evidence for the occurrence of bendable DNA region upstream of *rcsA* promoter through bioinformatics analyses 32. In this study, perhaps for the first time we have given the genetic evidence that supports the presence of putative curved DNA region upstream of *rcsA* promoter. Furthermore, we have shown that the H‐NS molecule which is defective in the formation of higher‐order oligomers can still function as a repressor at the *rcsA* promoter. The bioinformatics analyses show that the region around 400 bp upstream of *rcsA* promoter might serve as H‐NS binding site.

## Materials and methods

### Media composition, chemicals, fine chemicals and genetic and molecular techniques used in this study

The media (conventional LB and minimal media) composition used in this entire study is essentially as described in Ref. 33. Materials used for media, buffer, solutions, most of the antibiotics and other fine chemicals were purchased from HiMedia, India. Streptomycin was purchased from Sarabhai Chemicals, India, and the final concentration of each of them is quoted wherever appropriate. All the genetic techniques were according to Ref. 33 (with minor modifications), and molecular techniques employed in this study were as per Ref. 34.

### Bacterial strains and phages used in this study

Table 1 gives the list of bacterial strains, phages and plasmids used in this study. All the bacterial strains are the derivatives of *E. coli* K‐12, and the genetic nomenclature is according to Refs 35 and 36.

**Table 1:** List of bacterial strains/phages and plasmids used in this study

Strain	Relevant genotype	Source/reference/construction
SG20780	F^–^ *Δ(argF‐lac)169 lon510 cpsB10::lac rpsL150*	S Gottesman, NIH, USA
SG20781	F^–^ *Δ(argF‐lac)169 lon* ^+^ *cpsB10::lac rpsL150*	S Gottesman, NIH, USA
MG1655	F^–^ *rph‐1*	Laboratory collection
HR318	F^–^ λ^–^ *rph*‐1 *btuB::Tn10 rpoB8*	R. Harinarayanan, CDFD, India.
MGBT10	The same as MG1655, but has *btuB::Tn10*	This study, MG1655 X P1/(HR318)
MMRT6	The same as SG20780, but has *btuB::Tn10 rpoB12*	This study
MMRT23	The same as SG20780, but has *btuB::Tn10 rpoB77*	This study

Phage	Relevant genotype	Source/reference
P1	*Vir*	Laboratory collection, originally obtained from N Willets, UK

Name of the plasmid carrying *hns* variant alleles	Base change(s)/amino acid change present/functional defect(s) reported/reference	Source
pLG*hns*‐P116S	CCA to TCA Change of proline to serine at 116th amino acid position Shown to be defective in the recognition of curved DNA region, but retains nonspecific DNA binding [19, Ueguchi *et al*., 1996]	J Gowrishankar, CDFD, Hyderabad, India
pLG*hns‐∆64*	ATG to TGC (deletion of A results in a frameshift leading to formation of in‐frame Cys codon and a stop codon). Produces truncated H‐NS protein bearing only first 64 amino acids. Shown to be defective in DNA binding and higher‐order oligomerization [19, Ueguchi *et al*., 1996]	J Gowrishankar, CDFD, Hyderabad, India
pLG*hns*‐T55P	ACT to CCT Change of threonine to proline at 55th amino acid position Shown to be defective in higher‐order oligomerization [19, Ueguchi *et al*., 1996]	J Gowrishankar, CDFD, Hyderabad, India
pLG*hns*‐L26P	CTG to CCG Change of leucine to proline at 26th amino acid position Shown to be defective in higher‐order oligomerization [19, Ueguchi *et al*., 1996]	J Gowrishankar, CDFD, Hyderabad, India
pLG*hns* ^+^	Wild‐type DNA‐binding and oligomerization functions are normal (reviewed by Refs 8, 13)	J Gowrishankar, CDFD, Hyderabad, India

All the above‐mentioned plasmids are derivatives of pLG339 (pSC101 replicon, Kan^R^).

### Plasmid isolation, transformation and construction of strains bearing clones of dominant‐negative alleles of *hns*

The strains bearing the plasmids harbouring the dominant‐negative alleles of *hns*, namely pLG*hns*‐∆64, pLG*hns*‐P116S, pLG*hns*‐T55P, pLG*hns*‐L26P and pLG*hns* ^+^, were procured from J Gowrishankar, CDFD, Hyderabad, India. The plasmids were isolated from relevant strains using the alkaline lysis method 37, and the presence of insert was confirmed through restriction digestion analyses. The strains, namely SG20780 (∆*lon cps‐lac*), SG201781 (*lon* ^+^ *cps‐lac*), MMRT6 (∆*lon cps‐lac rpoB12*) and MMRT23 (∆*lon cps‐lac rpoB77*), were transformed with the relevant clones. The CaCl_2_‐mediated transformation technique was followed. As the vector backbone (pLG339) bears kanamycin as selection marker, the transformants were selected on LB plates containing kanamycin. Representative transformants from each case were selected and purified for further use.

### Beta‐galactosidase assay

0.1 mL of overnight cultures of each strain (carrying *cps‐lac* fusion) was subcultured into 5 mL of M9 minimal medium containing glucose as carbon source and grown at 30 °C. The cultures were allowed to attain mid‐log phase, and then, the optical density of the cultures was recorded at 600 nm wavelength. The beta‐galactosidase expressed from *cps‐lac* fusion was assayed as described in Ref. 33 with minor modifications.

### Bioinformatics analyses

The DNA sequence of the coding region of *rcsA* including the upstream region of *rcsA* promoter (till ‐600) was retrieved from Ecocyc.org, and the structure of the DNA sequence was elucidated using the software model.it (http://hydra.icgeb.trieste.it/dna/index.php). Further analyses/manipulations of the structure were carried out using pymol (http://pymol.org/edu/?q=educational).

## Results

### H‐NS which is defective in recognizing curved DNA fails to repress *rcsA* transcription

**Figure 1:**
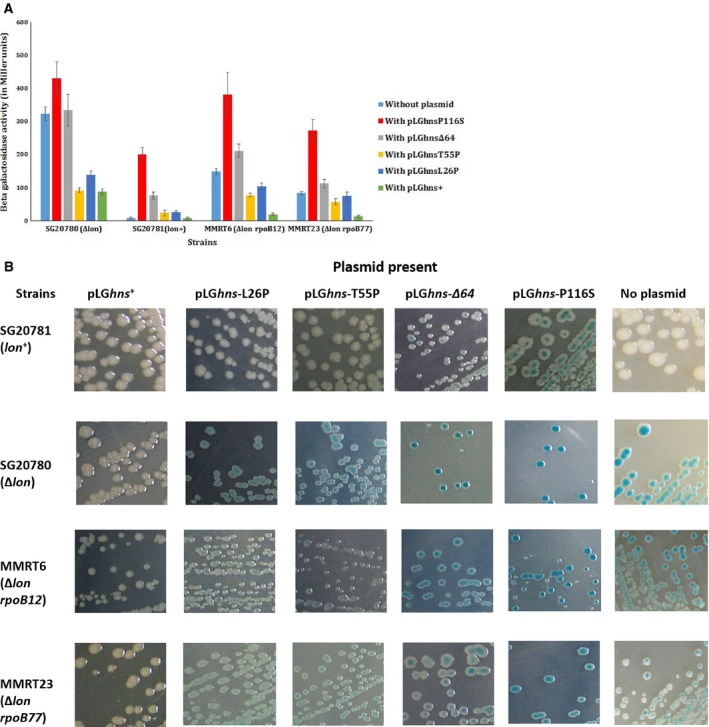
(A) Graphical representation of the expression pattern of *cps‐lac* fusion in relevant strains bearing clones harbouring dominant‐negative *hns* variant alleles, namely *hnsP116S, hnsL26P, hnsT55P* and *hns* ^+^. The β‐galactosidase experiments were performed seven times to minimize the error. The average ± SEM of values obtained from seven independent experiments is shown. (B) Pictures of sections of LB agar plates containing X‐gal (30 μg·mL^−1^) showing the Cps‐Lac phenotype of the relevant strains. All the strains were streaked and incubated at 30 °C for ~ 32 h. It is clear from the picture that the colonies of ∆*lon cps‐lac* strain SG20780 are in blue (Cps‐Lac^+^) and the colonies of *lon* ^+^ *cps‐lac* strain SG20781 are in white (Cps‐Lac^−^). For more details on expression pattern of Cps‐Lac fusion in relevant strains, refer to (A), and for actual values, refer to Table 2.

In our earlier study pertaining to the isolation and characterization of novel *rpoB* mutations capable of suppressing the overproduction of colanic acid Cps in *lon* mutant of *E. coli*, we have substantiated the role of functional H‐NS in the elicitation of Ces phenotype by the two *rpoB* mutations, namely *rpoB12* (C_**1576**_ to T_**1576**_; His526 to Tyr526) and *rpoB77* (C_**1535**_ to T_**1535**_; Ser512 to Tyr512) 32. As a continuation to this aspect, the effect of dominant‐negative alleles of *hns* in Ces strains (∆*lon rpoB12* and ∆*lon rpoB77*) and in parental strains (∆*lon* and *lon* ^+^) was studied (strains bearing the dominant‐negative *hns* alleles were procured from Gowrishankar, CDFD, India). For information of different dominant‐negative alleles of *hns* used in this study (Table 2). All the *hns* alleles, namely *hnsP116S*,* hns*∆*64*,* hnsT55P* and *hnsL26P*, have been cloned into a vector with its native promoter. The clone bearing *hns* ^+^ (pLG‐*hns* ^+^) was also used, as in this case the result could be presumed and it can be used for better comparison. All the clones were transformed into the relevant strains, namely SG20780 (∆*lon cps‐lac*), SG20781 (*lon* ^*+*^ *cps‐lac*), MMRT6 (∆*lon cps‐lac rpoB12*) and MMRT23 (∆*lon cps‐lac rpoB77*). In each case, a transformant was purified, cells were grown overnight in minimal glucose medium containing kanamycin till mid‐log phase and β‐galactosidase assay was carried out as described in Materials and methods. As was expected, introduction of the *hns* ^+^ clone reduced the expression of *cps‐lac* fusion to an appreciable degree in all the strains (Fig. 1). These results once again signify the role of H‐NS as a repressor of *rcsA* transcription. As shown in Fig. 1, introduction of clones bearing variant alleles of *hns*, namely pLG*hns*‐P116S and pLG*hns‐∆64*, into the relevant strains revealed the following: with reference to the mutant H‐NS (with P116S amino acid substitution) which is defective in binding to curved DNA, the expression level of *cps‐lac* has gone up to appreciable levels in all the above‐mentioned strains. Comparative analyses of the multicopy effect of *hns* ^+^ and *hnsP116S* alleles on the expression of *cps‐lac* fusion in the relevant strains clearly show the inability of the mutant form of H‐NSP116S molecules to exert complete repressor activity like the wild‐type although it is reported to retain nonspecific DNA‐binding activity (for the comparative values, see Table 2). These results strongly support the view that the upstream region of *rcsA* promoter is likely to contain a bendable/curved region and the inability of mutant form of H‐NS (H‐NSP116S) to bind to such putative bendable/curved region of *rcsA* promoter could be the cause for higher level of *cps‐lac* expression in the relevant strains. Introduction of pLG‐*hns∆64* increased the *cps‐lac* expression to some extent that clearly implies that the H‐NS molecules bearing only the N‐terminal region are not completely defective in repression at *rcsA* promoter.

**Table 2:** Summary of the multicopy effect of different dominant‐negative *hns* alleles on the level of expression of *cps‐lac* transcriptional fusion in the relevant strains and its implications on *rcsA* transcription

Strain/plasmid harbouring *hns* variant alleles and the levels of expression of β‐galactosidase from *cps‐lac* fusion (in Miller units) in the indicated strains. Values are average of seven different experiments		Inference on *rcsA* transcription based on *cps‐lac* expression
SG20780/pLG*hns*‐P116S	431	Introduction of pLG*hns*‐P116S significantly increased the *cps‐lac* expression in all the four strains. It is very clear that in the strains bearing pLG*hns*‐P116S, the expression level of *cps‐lac* is increased to an appreciable degree when compared to that of the strains bearing pLG‐*hns* ^+^ and relevant strains without any plasmid. These results clearly indicate that the mutant H‐NSP116S molecules could no longer serve as repressors for *rcsA* transcription. As H‐NSP116S molecules are reported to be defective in recognizing curved DNA region (although it retains nonspecific DNA‐binding activity), this observation leads to the inference that the upstream region of *rcsA* promoter should bear curved DNA region
SG20781/pLG*hns*‐P116S	200	
MMRT6/pLG*hns*‐P116S	382	
MMRT23/pLG*hns*‐P116S	272	
SG20780/pLG*hns‐∆64*	335	In the presence of pLG*hns‐∆64,* there is little increase in β‐galactosidase activity from *cps‐lac* fusion when compared to the *cps‐lac* expression in the respective strains bearing no plasmid. Although C‐terminally deleted H‐NS molecules are shown to have deficiency in DNA binding, they are reported to have more binding affinity towards chromosomally encoded wild‐type H‐NS molecules. As pLG*hns‐∆64* did not result in significant level of increase in *cps‐lac* expression, it suggests that binding of H‐NS∆64 with chromosomally encoded wild‐type H‐NS might probably help in retaining the repressor activity to some extent. However, when compared with the strains bearing pLG*hns* ^+^, there was a considerable elevation in the level of *cps‐lac* expression in all strains. These results signify the fact that although H‐NS∆64‐H‐NS^+^ hetero‐oligomers retain repressor activity, it perhaps cannot be equated to the activity of H‐NS^+^‐H‐NS^+^ homo‐oligomers
SG20781/pLG*hns‐∆64*	77	
MMRT6/pLG*hns‐∆64*	212	
MMRT23/pLG*hns‐∆64*	113	
SG20780/pLG*hns*‐T55P	92	Introduction of pLG*hns*‐T55P unexpectedly decreased the β‐galactosidase activity from *cps‐lac* fusion in all the strains to an appreciable degree. Comparison of the levels of expression of *cps‐lac* in the strains bearing pLG*hns*‐T55P with those of the relevant strains without plasmid clearly indicates the drastic reduction in the expression level of *cps‐lac* due to multicopy pLG*hns*‐T55P. It is possible when the oligomerization‐defective H‐NST55P molecules can still repress *rcsA* transcription, and it is perhaps due to nonspecific binding of the H‐NST55P molecules along the *rcsA* promoter region
SG20781/pLG*hns*‐T55P	24	
MMRT6/pLG*hns*‐T55P	77	
MMRT23/pLG*hns*‐T55P	58	
SG20780/pLG*hns*‐L26P	139	In a similar fashion to pLG*hns*‐T55P, introduction of pLG*hns*‐L26P also decreased the β‐galactosidase activity from *cps‐lac* fusion in the relevant strains. The inference and explanations could be the same as above (as in the case of pLG*hns*‐T55P)
SG20781/pLG*hns*‐L26P	26	
MMRT6/pLG*hns*‐L26P	104	
MMRT23/pLG*hns*‐L26P	75	
SG20780/pLG*hns* ^+^	88	Overexpression of the wild‐type functional H‐NS molecules represses the *rcsA* transcription much better as was expected
SG20781/pLG*hns* ^+^	9	
MMRT6/pLG*hns* ^+^	20	
MMRT23/pLG*hns* ^+^	14	
SG20780	323	As was expected in the absence of any clone, in the ∆*lon* strain a higher level of expression of *cps‐lac* was seen. However, in ∆*lon rpoB12* and ∆*lon rpoB77* mutants due to elicitation of Ces phenotype, the *cps‐lac* expression was less as was expected. In the *lon* ^+^ strain, in accordance with expectation, a very low level of expression of *cps‐lac* was seen due to RcsA degradation
SG20781	9	
MMRT6	149	
MMRT23	84	

### Oligomerization‐defective H‐NS can still function as a repressor at *rcsA* promoter

Structural analyses have revealed that the N‐terminal (amino acids 1–46) region of H‐NS is involved in the oligomerization 8. The clones, namely pLG*hns*L26P and pLG*hns*T55P, when introduced into the strains, namely SG20780 (∆*lon cps‐lac*), SG20781 (*lon* ^*+*^ *cps‐lac*), MMRT6 (∆*lon cps‐lac rpoB12*) and MMRT23 (∆*lon cps‐lac rpoB77*), surprisingly decreased the *cps‐lac* expression remarkably. The *hns* alleles cloned into these vectors result in the amino acid substitution at N‐terminal region (at amino acid positions T55P and L26P), which is expected to affect the oligomerization property of H‐NS molecules. This indirectly but strongly supports the view that even the oligomerization‐defective H‐NS could interfere with *cps‐lac* expression perhaps by repressing *rcsA* transcription (Fig. 1).

### Bioinformatics analyses reveal that H‐NS binding region could be present ~ 400 bp upstream of *rcsA* promoter

**Figure 2:**
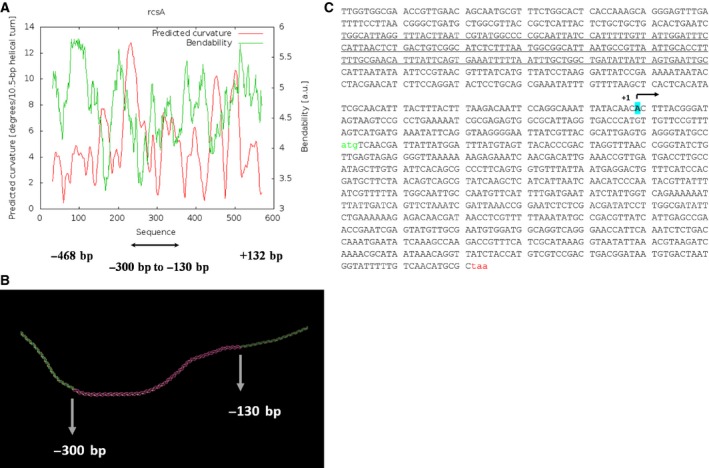
Bioinformatics analyses to show the bending nature of the DNA region present upstream of *rcsA* promoter. (A) Graphical representation to show the bending/curving nature of the upstream region of *rcsA* promoter. The software used for this analysis is bend.it, available at http://hydra.icgeb.trieste.it/dna/index.php. The base pair coordinate taken for this analysis is given below the figure. Shown in red is the bending ability, while green indicates the curving ability. (B) Using the software model.it, the curving/bending nature of the DNA region upstream of *rcsA* promoter is predicted. The region shown in red indicates the bending region corresponding to the base pair coordinate −130 to −300. Further analyses of the structure were carried out using PyMOL. (C) DNA sequence of *rcsA* gene was retrieved from Ecocyc.org, and the probable region for H‐NS binding has been predicted and is underlined.

H‐NS has been shown to bind to the curved DNA region preferentially 14, 15, 16. In our earlier report, we have given evidence for the involvement of functional H‐NS in the elicitation of Ces phenotype by *rpoB12* and *rpoB77* mutations, and we have predicted the presence of bendable DNA sequence upstream of the *rcsA* promoter 32. Here, we show that the DNA sequence around 400 bp upstream of the *rcsA* promoter is probably bendable in nature. Using the software model.it, we have modelled the DNA region present upstream of *rcsA*, and the image was further manipulated by pymol software. Figure 2A,B clearly shows that the region ‐130 to ‐400 does exist as a putative curved DNA. Therefore, the sequence underlined in Fig. 2C could be the most probable region where the H‐NS might bind to and repress *rcsA* transcription.

## Discussion

Overproduction of colanic acid Cps and extreme sensitivity to DNA‐damaging agents are considered as the iconic phenotypes of a *lon* mutant of *E. coli*. Detailed study on these aspects revealed that stabilization of two Lon substrates, namely RcsA (the positive regulator of *cps* transcription) and SulA (cell division inhibitor that gets induced upon DNA damage), is the main reason for the elicitation of the above‐mentioned phenotypes, respectively 38, 39, 40. Previous studies pertaining to isolation of suppressor(s) for these two hallmark phenotypes implicated a vital role of mutation in *ssrA* and a novel allele of *dnaJ* (*faa*) 41, 42, 43, 44. Earlier using an unorthodox, wee bit strategy, we sought for *rif* (*rpoB*) mutations capable of suppressing either one or both of the phenotypes of *lon* mutant. In such an attempt, we were indeed successful in isolating two such novel *rif* alleles (*rpoB12* and *rpoB77*) that could suppress only the overproduction of capsule synthesis. Detailed analyses showed that the elicitation of this Ces phenotype by these *rif* mutations primarily stems from the downregulation of *rcsA* transcription. Our study also revealed the requirement of functional H‐NS in the elicitation of Ces phenotype 32. Although the role of H‐NS in the transcriptional regulation of *rcsA* has been reported, the exact mode of regulation of *rcsA* transcription by H‐NS has not been reported to date.

Much of the information about the properties of different domains of H‐NS came from the analyses of effect of different mutations on the functionality of H‐NS. Systematic mutational analyses with H‐NS revealed that C‐terminal region is crucial for DNA binding and the central and N‐terminal regions are involved in the formation of oligomer/higher‐order oligomerization 8. During the course of such analyses, dominant‐negative variants of *hns* have been isolated 19, 20. In this study, the effect of different clones bearing dominant‐negative alleles of *hns* such as *hns*P116S, *hns*T55P, *hns*L26P, *hns*∆64 and *hns* ^*+*^, in phenotypically Ces strains such as MMRT6 (*∆lon rpoB12*) and MMRT23 (*∆lon rpoB77*) and also in ∆*lon* and *lon* ^+^ strains has clearly revealed that the *hnsP116S* allele that codes for H‐NS but is defective in recognizing curved DNA almost completely abolished the elicitation of Ces phenotype in both ∆*lon rpoB12* and ∆*lon rpoB77* strains. This effect was seen even in ∆*lon* and *lon* ^+^ strains. This indirectly implies that the region upstream of *rcsA* promoter might possess putative curvature which might play an important role in the regulation of *rcsA* transcription by H‐NS.

Further, the clones bearing *hns* alleles coding for the amino acid substitutions, namely T55P and L26P (which are defective in the formation of higher‐order oligomers), significantly reduced the *cps‐lac* expression not only in the *∆lon rpoB12* and *∆lon rpoB77* strains but also in the ∆*lon* and *lon* ^+^ strains; that is, the effect is albeit closer to that of wild‐type H‐NS. This was totally unexpected as we imagined that the mutant forms of H‐NS cannot form higher‐order oligomers and therefore will not be able to repress *rcsA* transcription. But the fact that we have made such an observation compelled us to make a model that in a nonoligomeric state and even without forming higher‐order oligomers, these mutant forms of H‐NS perhaps might be able to bind to DNA and function as repressors for *rcsA* transcription. Williams *et al*. 19 have reported that the introduction of clone(s) bearing oligomerization‐defective *hns* alleles, namely L26P and T55P, drastically decreased the expression of semisynthetic *5A6Agal* promoter. Similar analyses with one other H‐NS‐regulated gene, namely *proU*, indicate that the expression of its promoter was not found to be identical to that of *5A6Agal* promoter. These observations signify the fact that the regulatory function of H‐NS depends on the sequence features of the promoters also. In similar analyses, it was also found that introduction of clone bearing *hns* allele (P116S) elevated the expression of *5A6Agal* promoter to an appreciable degree, while the same was once again not found to be true with *proU* promoter. It has been reported that the upstream region of *5A6Agal* promoter bears a curvature 45. However, in the case of *proU*, the presence of any curved DNA region has not been reported and notably the repression by H‐NS essentially needs extensive nucleoprotein formation at the *proU* promoter 45, 46, which perhaps gives a clue about H‐NS binding‐induced DNA bending at the *proU* promoter region.

The expression pattern of *5A6Agal* promoter and *rcsA* promoter is found to be similar in the presence of different dominant‐negative *hns* alleles. These observations clearly favour the notion that the upstream region of *rcsA* might possess curved DNA which would serve as binding region for H‐NS, thus aiding H‐NS to transcriptionally regulate *rcsA* expression.

## Author contributions

SM designed the study, performed experiments, analysed the results and wrote the manuscript. MK performed the experiments. MHM analysed the results, wrote the manuscript and provided resources for the study.
